# MiRNA182 regulates percentage of myeloid and erythroid cells in chronic myeloid leukemia

**DOI:** 10.1038/cddis.2016.471

**Published:** 2017-01-12

**Authors:** Deepak Arya, Sasikala P Sachithanandan, Cecil Ross, Dasaradhi Palakodeti, Shang Li, Sudhir Krishna

**Affiliations:** 1 Cellular Organization and Signalling Group, National Centre for Biological Sciences, Tata Institute of Fundamental Research, Bangalore, India; 2 Manipal University, Manipal, India; 3 Department of Medicine, St Johns Medical College and Hospitals, Bangalore, India; 4 Stem Cells and Regeneration Group, Institute for Stem Cell Biology and Regenerative Medicine, Bangalore, India; 5 Duke-NUS Graduate Medical School, Singapore

## Abstract

The deregulation of lineage control programs is often associated with the progression of haematological malignancies. The molecular regulators of lineage choices in the context of tyrosine kinase inhibitor (TKI) resistance remain poorly understood in chronic myeloid leukemia (CML). To find a potential molecular regulator contributing to lineage distribution and TKI resistance, we undertook an RNA-sequencing approach for identifying microRNAs (miRNAs). Following an unbiased screen, elevated miRNA182-5p levels were detected in Bcr-Abl-inhibited K562 cells (CML blast crisis cell line) and in a panel of CML patients. Earlier, miRNA182-5p upregulation was reported in several solid tumours and haematological malignancies. We undertook a strategy involving transient modulation and CRISPR/Cas9 (clustered regularly interspersed short palindromic repeats)-mediated knockout of the *MIR182* locus in CML cells. The lineage contribution was assessed by methylcellulose colony formation assay. The transient modulation of miRNA182-5p revealed a biased phenotype. Strikingly, *Δ182* cells (homozygous deletion of *MIR182* locus) produced a marked shift in lineage distribution. The phenotype was rescued by ectopic expression of miRNA182-5p in *Δ182* cells. A bioinformatic analysis and Hes1 modulation data suggested that Hes1 could be a putative target of miRNA182-5p. A reciprocal relationship between miRNA182-5p and Hes1 was seen in the context of TK inhibition. In conclusion, we reveal a key role for miRNA182-5p in restricting the myeloid development of leukemic cells. We propose that the *Δ182* cell line will be valuable in designing experiments for next-generation pharmacological interventions.

The pathways that regulate haematopoietic differentiation are well understood and have served as paradigms in developmental biology.^1^ With the discovery of microRNAs (miRNAs), there has been an interest in analysing the role of these molecules in haematopoiesis and related disease states.^1, 2, 3, 4^ Examples of such miRNAs are miRNA223, miRNA486, miRNA144 and miRNA451.^6, 7^ Specifically, in the context of hematopoietic development and malignancies, a miRNA of particular interest is miRNA182-5p.^5, 6, 7, 8, 9^ The locus that encodes miRNA182 is located on chromosome 7q32.2 of human genome in a cluster of three miRNAs—*MIR182*, *MIR183* and *MIR96*.^13^ The miRNA182 locus has been reported to be abundantly expressed in the retina, central nervous system and normal human embryonic stem cells.^14^ In hematopoietic development, miRNA182-5p has been shown to regulate clonal expansion of T cells.^15^ Although miRNA182-5p is clearly of importance in cancer progression and therapeutic resistance,^9, 13, 16^ its role in lineage distribution remains uncharacterized.

Chronic myeloid leukemia (CML), with its well-defined genetic characterization, has lent itself to a molecular understanding of cancer progression.^17, 18^ The disease progression is invariably associated with a Philadelphia translocation or 9–22 chromosomal rearrangement, the product of which is an activated tyrosine kinase, Bcr-Abl.^19^ It has been reported that Bcr-Abl promotes proliferation of multipotent progenitors and myeloid progenitor cells while suppressing erythroid progenitors.^20^ Over the past decade or so, a considerable body of literature has documented the striking clinical remissions that Gleevec and next-generation tyrosine kinase inhibitor (TKI) have achieved.^21, 22, 23^ However, there is a small but significant set of CML patients who develop resistance to TKIs in the absence of specific identifiable tyrosine kinase mutations.^24, 25^ The mechanisms associated with this class of relapse are currently poorly understood.

The reciprocal relationship between proliferation and differentiation determines the steady-state distribution of subsets of hematopoietic cells.^26, 27^ The two major classes of these subsets are erythroid and myeloid-derived cells. The erythroid class encompasses colony-forming unit (CFU)—megakaryocyte cells (Meg), an intermediate erythroid progenitor—burst-forming unit erythroid (BFU-E) and CFU-E.^28^ The myeloid compartment includes CFU-Granulocyte, CFU-Macrophage and CFU-GM (granulocyte–macrophage).^29^ In the absence of a disease, measurements of steady states typically reveal a low percentage (<5%) of myeloid blast cells in the bone marrow.^21, 30^ In contrast, myeloid leukemias are characterized by a high percentage of myeloid blast cells (upto 50%).^31^ Similarly, erythroid leukemic conditions display a higher percentage of erythroid blasts.^32^ A paradoxical observation is the detection of an erythroid burden in the late-stage CML patients.^21^

The mechanisms of altered expression of miRNA182 in myeloid leukemia remain poorly characterized. With an interest in delineating the role of miRNAs that regulate TKI resistance, we used K562 cells; a multipotent Bcr-Abl-positive CML cell line.^33^ We undertook a small RNA-sequencing analysis to identify the miRNAs that were potentially upregulated in the context of TKI resistance. We found a striking association between elevated miRNA182-5p level and TKI resistance in CML lines and primary cells. The colony formation assay determined that ectopic miRNA182-5p expression is positively correlated with erythroid differentiation. We embarked on a precise gene-editing approach using CRISPR (clustered regularly interspaced short palindromic repeats) and generated *Δ182* cells. The loss of miRNA182 expression by both locked nucleic acid (LNA) anti-miRNA and CRISPR knockout revealed an increase in myeloid differentiation. Further, we examined a role for Hes1, a putative target of miRNA182-5p in regulating percentage of myeloid and erythroid cells (ME%). Collectively, elevated miRNA182-5p expression blocked the myeloid differentiation of K562 cells. This study deciphers the role of miRNA182-5p in a conserved lineage program of leukemic cells and holds promise to the use of miRNA182-5p for therapeutic improvements in parallel to TKI therapy.

## Results

### High miRNA182-5p expression is associated with TKI resistance in CML cells

**Figure 1:**
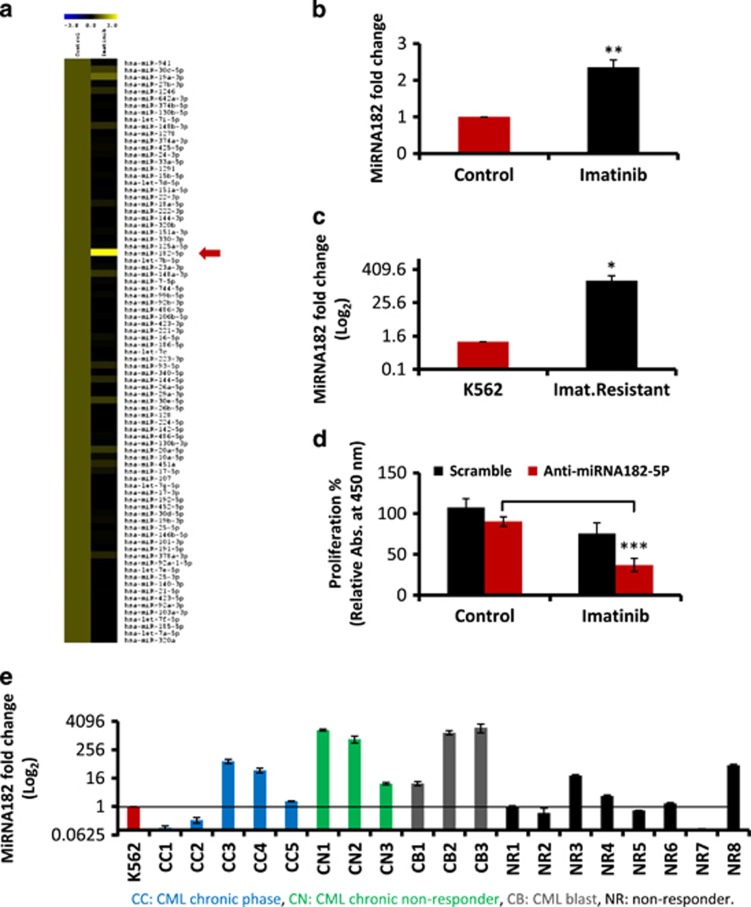
High expression of MiRNA182-5p is associated with TK inhibitor resistance in CML cells. (**a**) Heatmap of differentially expressed miRNAs between control and imatinib-treated K562 cells. Column labels represent the type of sample: control and imatinib. The red arrow shows miRNA182-5p expression in the heatmap. Range of expression measured was −3- to +3-fold change. (**b** and **c**) Expression of miRNA182-5p in K562 cells after imatinib treatment (**b**) and imatinib-resistant K562 cells (**c**). Data are shown as mean of three independent experiments. Error bars show s.e. of three independent experiments with *P*-values of the mean. **P<0.01 and ***P<0.001. (**d**) Percentage cell proliferation of LNA anti-miRNA182-5p-transfected K562 cells with TK inhibitor. The data are compared with scramble control. Data are shown as mean of three independent experiments. Error bars show s.e. of three independent experiments with *P*-values of the mean. *P<0.05, ***P<0.001. (**e**) Expression of miRNA182-5p in clinical samples of CML. The *Y*-axis shows the fold change in miRNA182-5p expression (log_2_ scale). Data are presented as mean of three biological triplicates. The normalisation is undertaken to the expression of miRNA182-5p from K562 cell line. CC, CML chronic phase; CN, CML chronic nonresponder; CB, CML blast; NR, nonresponder

To assess the miRNAs that were modulated in the context of resistance to imatinib, Illumina sequencing was performed on RNA extracted from imatinib-treated K562 cells. The K562 cell line retains a rearranged Bcr-Abl gene, with no detectable mutations. Further, this cell line can be induced to differentiate and thus serves as a model for analysing the contribution of distinct lineages to late-stage CML progression.^33, 34^ In Figure 1a, we showed the expression profile of all the miRNAs from imatinib-treated K562 cells compared with an online available data set from untreated K562 cells (courtesy Professor Alok Bhattacharya, JNU).^35^ The heatmap revealed that the expression of 83 miRNAs was altered (Supplementary Table 2). Of particular interest was the detection of a twofold increase in miRNA182-5p expression (Figure 1a). Quantitative PCR analysis of miRNA182-5p revealed a twofold increase in both K562 cells (Figure 1b) and KCL22 cells (Supplementary Figure S2A). There was a 160-fold increase of miRNA182-5p expression in K562 cells resistant to imatinib (Figure 1c).

To directly explore the role of miRNA182-5p expression in TKI resistance, we used LNA anti-miRNAs in cell proliferation assay. The anti-miRNA182-5p and scramble transfection revealed 37% and 75% rate of proliferation, respectively, after imatinib treatment (Figure 1d). A marked 10-fold increase in the expression of miRNA182 was noted in six CML samples of early nonresponders and late progressors. Out of the 19 samples, three nonresponders revealed an increase in the expression of miRNA182. These data were generated in comparison with miRNA182 expression in K562 cells (Figure 1e).

The results in Figure 1 suggest an association of miRNA182-5p expression with TKI resistance in CML, particularly in either the later stages of the disease or early nonresponders. Given the contribution of miRNAs in differentiation and development,^36, 37^ their causality with chemoresistance by modulating the differentiation pathways remain unexplored. To assess the role of miRNA182-5p in lineage distribution, we undertook experiments to overexpress or downregulate miRNA182-5p expression in K562 cells.

### MiRNA182-5p modulation and TKI resistance are associated with shift in ME%

To determine whether K562 cells with transient miRNA182-5p perturbation retained their potential to differentiate into erythroid and myeloid cells, we performed methylcellulose colony-forming assay. The percentage of erythroid and myeloid colonies was measured. We assessed the lineage distribution of K562 cells in the context of TKI resistance by treating K562 cells with 0.75 *μ*M concentration of imatinib. The mean number of colonies of BFU-E were 61, 53 and 29, CFU-G were 10, 8 and 1, CFU-E were 16, 11 and 1, and total colony counts were 93, 72, and 33 in RPMI-, DMSO- and imatinib-treated K562 cells, respectively (Supplementary Figure 9e). The graphical representation of this data shown in Supplementary Figure 9f revealed a marginal increase of erythroid cells % (88% *versus* 93%) in imatinib-treated K562 cells.

**Figure 2:**
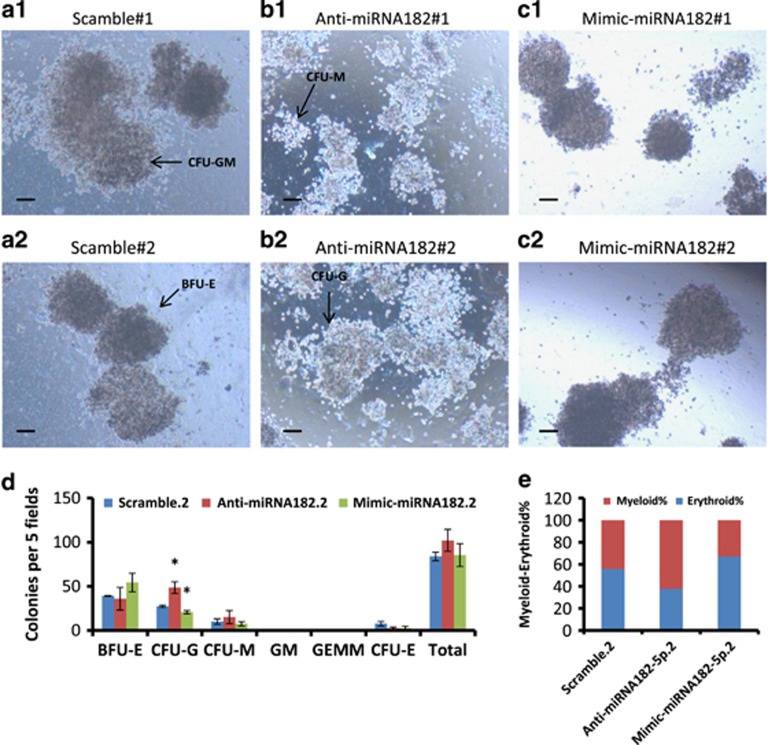
Modulation in the expression of MiRNA182-5p results in a shift of ME% in K562 cells. (**a**–**c**) Representative images of colonies formed in methylcellulose CFU assay with scramble- (**a**), anti-miRNA182-5p- (**b**) and miRNA182-5p mimics-transfected K562 cells (**c**). Images are taken at × 4 magnification. Scale bar represents 100 *μ*m. BFU-E, CFU-M, CFU-G and CFU-GM are shown in a2, b1, b2 and a1, respectively. (**d**) Quantitation of the colony counts and types on day 21 with each condition. Data are shown as number of colonies per five fields. Error bars show s.e. of three independent experiments with *P*-values of the mean. *P<0.05, **P<0.01 and ***P<0.001. (**e**) ME% shows the lineage distribution in colony formation assay

Next, to determine the lineage distribution of K562 cells after miRNA182 modulation, we used anti-miRNA182 and mimics-miRNA182 on K562 cells. The mean number of colonies of BFU-E were 39, 36 and 54, CFU-G were 27, 48 and 21, CFU-M were 10, 15 and 7 in Scramble, LNA anti-miRNA182-5p- and mimics-miRNA182-transfected K562 cells, respectively (Figure 2d). The graphical representation of this data shown in Supplementary Figure 2e revealed an increase and decrease in ME% (62% and 33% *versus* 44%) in LNA anti-miRNA182-5p- and mimics-miRNA182-transfected K562 cells, respectively. The quantitative data for the all the colony types in each condition were provided Figure 2d.

The data from Figure 2 and Supplementary Figure 9 suggested that miRNA182-5p expression might augment TKI resistance by altering the lineage distribution. The results in Figure 2 were found analogous to that seen in Supplementary Figure 7. To generate direct evidence that miRNA182-5p regulates ME%, *MIR182* genomic locus was deleted using the CRISPR system.

### Generation of CRISPR-based knockout system for *MIR182* locus

**Figure 3:**
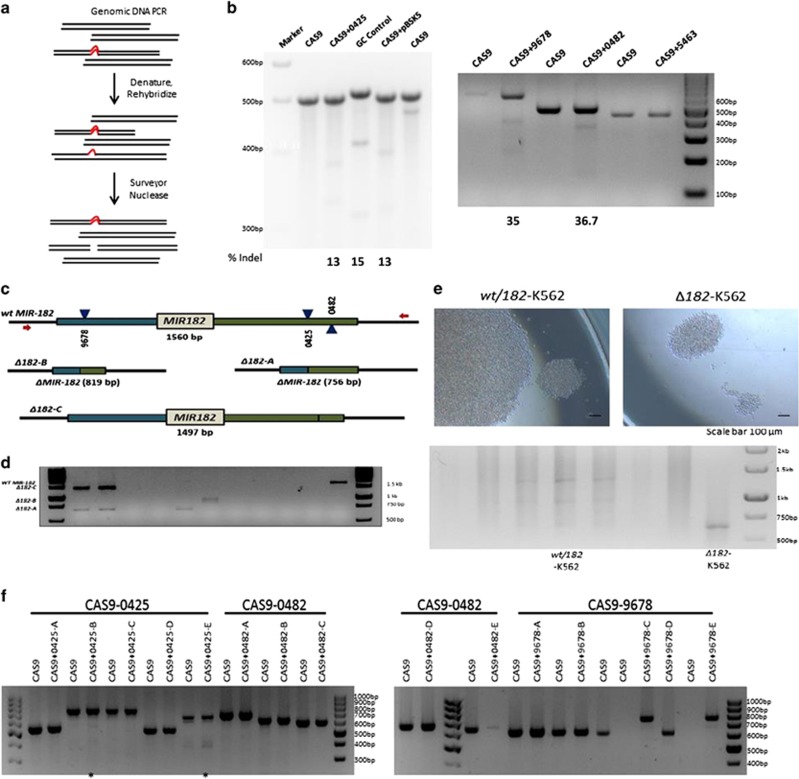
Generation of a CRISPR-based knockout system to delete *MIR182* locus. (**a**) A Cartoon diagram shows the surveyor nuclease assay. (**b**) Surveyor nuclease assay of each guide RNAs targeting *MIR182* locus in HEK293T cells. Percentage indels are shown under each guideRNA. (**c**) Schematic of three guideRNA approach for targeting *MIR182* locus in K562 cells. CAS9+9678, CAS9+0482 and CAS9+0425 are used to delete *MIR182* genomic locus from K562 cells. (**d**) Agarose gel electrophoresis shows PCR amplification of *MIR182* locus in CRISPR guide RNAs nucleofected K562 cells. *wtMIR182* (1560 bp), *Δ182*-A (756 bp), *Δ182*-B (819 bp), *Δ182*-C (1497 bp). (**e**) Colonies from *Δ182* and *wt/182* cells grown in a 96-well plate as single cells. Images are taken at 10 days. Scale bar represents 100 *μ*m. (**f**) Agarose gel analysis shows off-target sites of CRISPR guide RNAs targeting *MIR182* locus by surveyor nuclease assay. The 0425-E site shows additional bands

**Figure 4:**
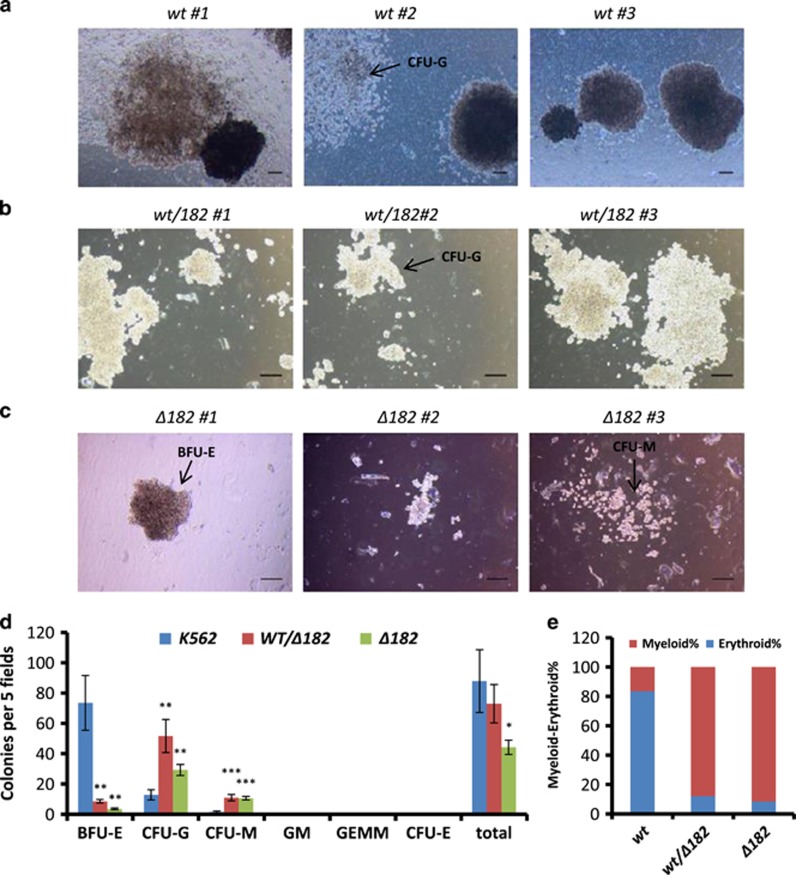
*MIR182* deletion markedly shifts ME% in K562 cells. (**a**–**c**) Representative images of colonies formed in methylcellulose CFU assay with *wt* (**a**), *wt/182* (**b**) and *Δ182* cells (**c**), respectively. Images are taken at × 4 magnification. Scale bar represents 100 *μ*m. CFU-Gs are shown in a2 and b2. BFU-E and CFU-M are shown in c1 and c3, respectively. (**d**) Quantitation of the colony counts on day 21 with each condition. Data are shown as number of colonies per five fields. Error bars show s.e. of three independent experiments with *P*-values of the mean. *P<0.05, **P<0.01 and ***P<0.001. (**e**) ME% shows the lineage distribution in colony formation assay

CRISPR/Cas9 system has been used to modify genomic loci of clinical importance.^38^ A stepwise process to delete the *MIR182* locus was undertaken starting with HEK293 to standardise knockout method (Supplementary Figure 5a). To reduce the incidence of off-target effects, three CRISPR plasmids (targeting 129409678, 129410425 and 129410482 according to GRCh37.p13 of human genome assembly) were nucleofected in K562 cell line (Figure 3c). Multiple clones with precise deletion (819 bp, 756 bp and 1497 bp) were detected by PCR amplification of *MIR182* locus followed by agarose gel electrophoresis (Figure 3d). To further confirm whether the amplified products corresponded to the *MIR182* locus, Sanger sequencing was performed on the purified PCR products. The chromatogram confirmed that *MIR182* locus was deleted (Supplementary Figure 6b). *MIR182* deleted clones were used to make heterozygous (*wt/182*) and homozygous lines (*Δ182*) for further study (Figure 3e). We quantified miRNA182 expression levels by qPCR in K562 cells after *MIR182* deletion. There was a significant reduction in miRNA182-5p levels of 66% and 62% in *wt/182* and *Δ182* cells, respectively, compared with wild-type K562 cells (Supplementary Figure 10g). To assess the proliferation potential of K562 cells after deletion of *MIR182* locus, we used WST1 cell proliferation assay. The *Δ182* cells and K562 cells displayed 23% and 100% proliferation rate, respectively (Supplementary Figure 10h). The cell proliferation data were analogous to the imaging data seen in Figure 3e. The cumulative results in Figures 3 and 4 and Supplementary Figure 10 revealed that the CRISPR system efficiently deleted the *MIR182* locus in K562 cells and a decrease in the rate of proliferation was detected in these cells.

### *MIR182* locus contributes to the maintenance of ME% in K562 cells

As an inverse relationship between proliferation and differentiation had been predicted earlier,^39^ we assessed whether the loss of *MIR182* locus resulted in any specific lineage commitment. The mean number of colonies of BFU-E were 73, 8 and 4, CFU-G were 13, 52 and 29, CFU-M were 1.5, 11 and 10, and total colonies were 88, 73 and 44 in K562, *wt/182* and *Δ182* cells, respectively (Figure 4d). The graphical representation of this data shown in supplementary Figure 4e revealed an increase of ME% (88% and 92% *versus* 16%) in *wt/182* and *Δ182* cells, respectively.

**Figure 5:**
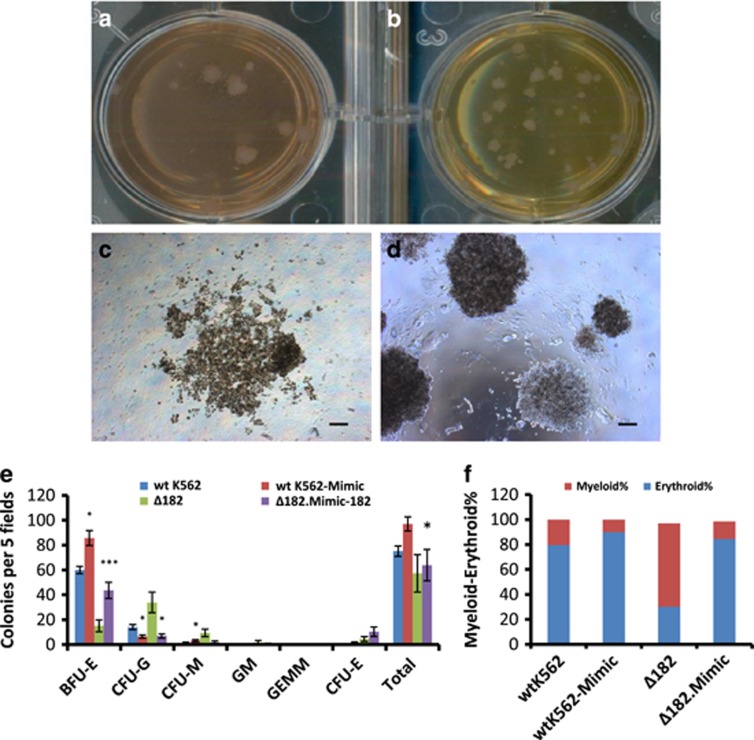
Ectopic expression of miRNA182-5p increases erythroid phenotype in *Δ182* cells. (**a** and **d**) An image of a Petri plate showing the colonies of *Δ182* cells (**a**) and mimics-miRNA182-5p-transfected *Δ182* cells (**b**). A magnified region of the same is shown in **c** and **d**. (**e**) Quantitation of the colony counts on day 21 with each condition. Data are shown as number of colonies per five fields. Error bars show s.e. of three independent experiments with *P*-values of the mean. *P<0.05, **P<0.01 and ***P<0.001. ME% shows the lineage distribution in colony formation assay

To exclude the possibility of off-targets contributing to lineage shift, miRNA182-5p mimics were transfected in *Δ182* cells. The mean number of colonies of BFU-E were 15 and 43, CFU-G were 34 and 7, CFU-M were 10 and 2, and total colonies were 57 and 64 in *Δ182* cells and miRNA182-5p mimics-transfected *Δ182* cells, respectively (Figure 5e). The graphical representation of this data shown in supplementary Figure 5f revealed an increase of ME% (67% *versus* 14%) in miRNA182-5p mimics-transfected *Δ182* cells, respectively (Supplementary Figure 10I and Figure 5f).

Figures 4 and 5 showed that *Δ182* cells became poised for myeloid differentiation and the phenotype was rescued by ectopic expression of miRNA182-5p. The data from *MIR182* knockout were found analogous to that seen in Figure 2. Collectively, Figures 1,2,3,4 and 5 revealed that lineage distribution of K562 cells was associated with TKI resistance. In addition, miRNA182-5p played a central role in determining the lineage distribution. To strengthen the role of miRNA182-5p in above context, we queried for intersecting pathways of miRNA182-5p signalling.

### Hes1 serves as a putative target of miRNA182-5p in regulating ME%

**Figure 6:**
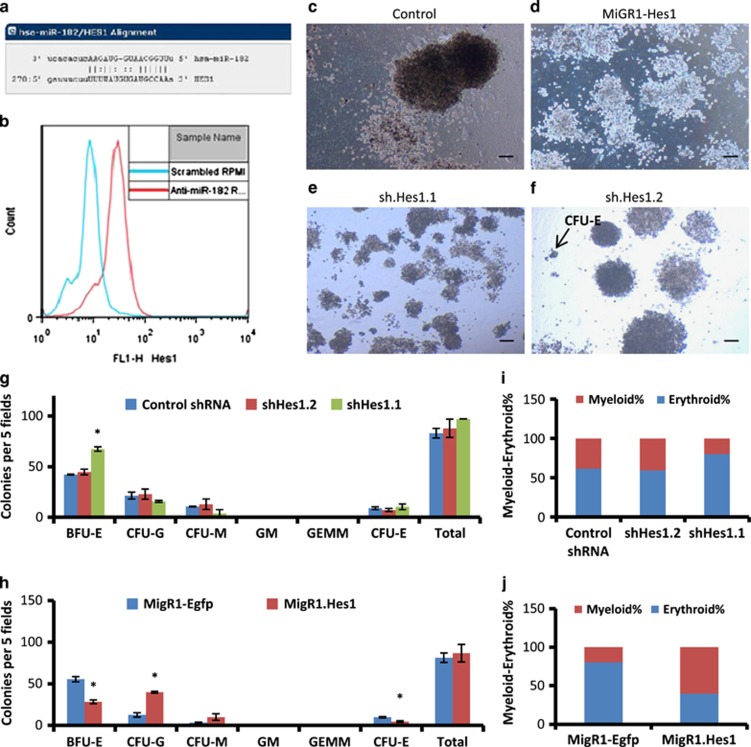
Hes1 serves as a putative target of miRNA182-5p in regulating ME%. Human Hes1 has a 7-mer conserved binding site at its 3′-UTR for miRNA182-5p. (**a**) Flow cytometric analysis of Hes1 expression in anti-miRNA182-5p-transfected K562 cells. The blue and red histograms represent Hes1 expression in scramble control and miRNA182-5p inhibition, respectively. **(c**–**f**) Representative images from the colony formation assay in K562 cells (**c**), miGR1-Hes1- (**d**), shRNA1-Hes1- (**e**) and shRNA2-Hes1-nucleofected K562 cells (**f**), respectively. Images are taken at × 4 magnification. Scale bar represents 100 *μ*m. (**g** and **h**) Quantitation of the colony counts from the shRNA- (**g**) and MiGR1-Hes1-nucleofected K562 cells (**h**) on day 21. Data are shown as number of colonies per five fields. Error bars show s.e. of three independent experiments with *P*-values of the mean. *P<0.05, **P<0.01 and ***P<0.001. (**i** and **j**) ME% showing percentage of myeloid and erythroid cells in colony formation assay from shRNA- and MiGR1-Hes1-nucleofected K562 cells

The miRNA-Org, DIANA-microT and target-scan tools predicted a 7-mer conserved binding site for miRNA182-5p on 3′-UTR of Hes1 mRNA (Figure 6a). To directly explore this interaction, anti-miRNA182-5p were transfected in K562 cells and Hes1 expression was quantified by flow cytometry. The histogram showed an increase in Hes1 expression in the anti-miRNA182-5p-transfected cells compared with scramble-transfected K562 cells (Figure 6b). To address the role of Hes1 in lineage distribution, we undertook a loss and gain of function approach. The mean number of colonies of BFU-E were 42 and 67, CFU-G were 21 and 16, CFU-M were 10 and 4, CFU-E were 9 and 10 in control shRNA- and shHes1.1-nucleofected K562 cells, respectively (Figure 6g). The mean number of colonies of BFU-E were 55 and 28, CFU-G were 13 and 40, and CFU-M were 3 and 10 in miGR1-eGFP- and miGR1-Hes1-nucleofected K562 cells, respectively (Figure 6h). The graphical representation of this data shown in Figures 6i and j revealed a decrease and increase of ME% (20% and 60% *versus* 14%) in shHes1.1- and miGR1-Hes1-nucleofected K562 cells, respectively.

In a translational approach, Hes1 mRNA expression levels were assessed in primary CML cells. The qPCR analysis showed an increase in Hes1 expression in three out of seven cases after anti-miRNA182-5p transfection (Supplementary Figure 4). Collectively, Hes1 served as a putative target of miRNA182-5p in CML and affected the lineage distribution of CML cells. These results were analogous to that seen in Figures 2 and 4.

#### Extended results

A bioinformatic approach was applied on miRNA-sequencing data to examine miRNA182-5p signalling pathway (Supplementary Figure 3a). The sequencing data revealed an upregulation in expression of miRNA182-5p and miRNA19. In addition, 81 other miRNAs were downregulated in imatinib-treated K562 cells (Supplementary Table 2). These miRNAs were examined for their putative pathways by miRNA-path tool from Diana lab. The pathway analysis predicted TGF-β signalling as one of the candidate pathways upstream to miRNA182-5p regulation (Supplementary Figure 3b). Earlier, inhibition of TGF-β signalling has been shown to downregulate the colony formation ability of leukemia initiating cells.^40^ To find a direct link between TGF-β and miRNA182 expression, K562 cells were treated with recombinant TGF-β ligand. The treatment resulted in 1.3- and 5.8-fold increase in miRNA182-5p expression with 2 ng and 5 ng ligand concentration, respectively (Supplementary Figure 3c). The 10 ng concentration treatment resulted in the decrease of miRNA182-5p levels to 0.45-fold (Supplementary Figure 3c). Next, a link between TK resistance and TGF-β was explored. The TKI-treated K562 cells showed a marginal increase in TGF-β receptor (Supplementary Figure 3d) and p-Smad3 levels (Supplementary Figure 3e) at the protein level.

## Discussion

**Figure 7:**
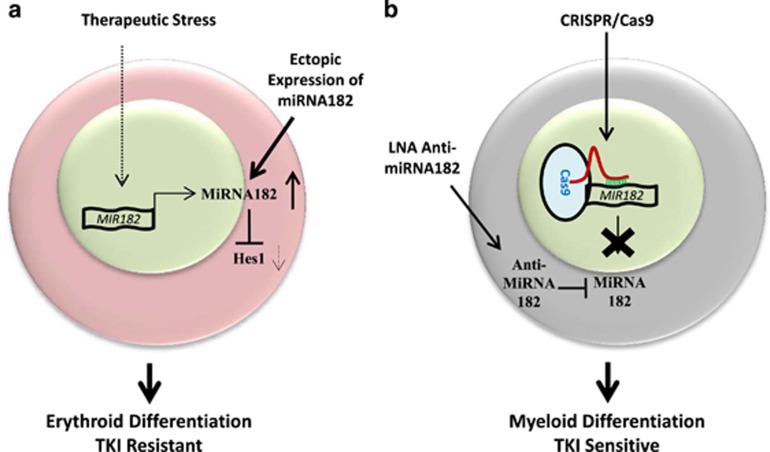
MiRNA182-5p-mediated control over differentiation program contributes to TKI resistance of CML cells. (**a**) A cartoon diagram representing the mechanisms of miRNA182-mediated erythroid differentiation in K562 cells. The TK inhibitor induces the overexpression of miRNA182-5p that results in suppression of Hes1 expression. The cells acquire erythroid phenotype and become TKI resistant. Ectopically overexpressed miRNA182-5p leads to erythroid differentiation of K562 cells. The dotted arrows show correlation based on the expression. The solid arrows show direct interactions. (**b**) A cartoon diagram representing the myeloid differentiation in K562 cells mediated by the loss of miRNA182-5p expression. First, CRISPR system targeting *MIR182* locus abrogates miRNA182-5p expression. This leads to myeloid differentiation of K562 cells. The transfection of anti-miRNA182-5p in K562 cells also induces a myeloid-biased phenotype and TKI sensitivity

In this study, we examined the patterns and consequences of the expression of miRNA182-5p in the context of CML progression. The expression patterns revealed that the expression of miRNA182-5p was associated with TKI resistance in CML cells. The experiments involving lowering the expression of miRNA182-5p resulted in the change of lineage distribution of K562 cells with an enhanced detection of myeloid cells (Figure 7b). We also noted that the expression of miRNA182-5p correlated with the proliferation potential of CML cells. Collectively, these data suggested that miRNA182-5p upregulation concomitantly contributed to both accumulation of erythroid cells as well as resistance to TKIs (Figure 7a).

Our experimental approach was built on an initial RNA-sequencing data that revealed a twofold increase in miRNA182-5p levels in the context of TKI resistance in a cell line model (Figure 1a). In 6 out of 19 cases, the linkage of TK resistance with miRNA182-5p levels was noted in the clinical context of late progressing CML cases. Thus, miRNA182-5p expression could be a major contributor to late state progression, given the heterogeneity of mechanisms that drive progression at this stage.^41^ These findings correlated with the patterns of miRNA182-5p expression identified in the context of chemoresistance in other tumors.^42, 43, 44^ The data from Figure 1 and Supplementary Figure 1 suggested a miRNA182-5p-dependent TKI resistance mechanism independent of Bcr-Abl. Previously, a TGF-β/FOXO signalling was suggested to be contributing to TKI resistance and EMT transition seen in CML stem cells.^40, 45, 46^ Wnt signalling that has been implicated in CML stem cells also shown to be upstream regulator of expression of miRNA182.^47, 48^ Given the diversity of regulatory signalling pathways upstream and downstream to miRNA182, different stages of CML might require rewiring of the miRNA182-dependent transcriptional programs. The development of modulators of differentiation might synergise with other therapeutic strategies in the management of late-stage CML.

Two independent studies suggested that the outcomes of TKI therapy might be linked with increased miRNA182-5p levels and a differentiation imbalance.^45, 49, 50^ The erythroid-biased phenotype in a steady-state condition was increased by ectopic expression of miRNA182-5p in a methylcellulose assay (Figure 2). The ectopic expression might overestimate the contribution of miRNA182-5p expression in lineage decisions. Thus, we used an approach that generated loss of physiological expression of miRNA182-5p by both transient and constitutive means. The data from loss of physiological expression using LNA anti-miRNAs were strengthened by CRISPR-mediated complete loss of miRNA182-5p expression (Figure 5). In the current study, we showed that the differentiation program might contribute to TKI resistance and miRNA182-5p expression was deterministic of the process (Figures 1,2,3,4 and 5). The assay performed on K562 cells that displayed limited differentiation potential could be improved by addressing the question in early progenitor cells such as hematopoietic stem cell and using an animal model to track the cells for a longer time period.

The data from Figure 6 revealed that Hes1 expression was rescued by the loss of miRNA182-5p levels. In addition, perturbation in Hes1 levels resulted in an erythroid–myeloid imbalance in K562 cells. Hematopoietic factors such as GRB2, Rac1 and CEBPA, and apoptotic factors such as FOXO1, PDCD4 and BCL2, respectively, have been shown to be regulated by miRNA182.^51, 52, 53^ Given the unresolved regulation of Hes1 in myeloid differentiation and leukemias,^54, 55^ we provided evidence for a post-transcriptional regulatory mechanism in Notch signalling pathway mediated by miRNA182. The data generated in our study would suggest a rethinking of the role of miRNA182 signalling in the context of late-stage CML and particularly TKI resistance.

The use of both the cell line that we have generated and clinical analysis should offer an opportunity to create novel therapies for the relapsing CML cases in the years ahead. As a wider implication, we also validate that gene editing of specific locus in established cell lines is a useful approach to create reagents for phenotypic analysis in the context of drug resistance.

## Materials and methods

### Cell culture

K562 and KCL22 cells were obtained from Dr. Soumen Chakraborty lab (ILS, Bhubaneswar, India). The cells were cultured in 10% RPMI media with foetal calf serum (v/v) and 0.1 mg/ml PenStrep at 37 °C and 5% CO_2_ conditions. Cells were passaged every 2–3 days and used for study till passage 20. The cells were tested for mycoplasma routinely and found negative consistently. Short-term TKI treatment was performed at inhibitory concentration 50 (IC_50_) −0.75 *μ*M of imatinib Mesylate (purchased from Santa Cruz Biotechnology, Santa Cruz, CA, USA) and incubated for 48 h. The dead cells were washed and live cells were scored for Bcr-Abl activity by measuring the expression of p-Crkl, a downstream target molecule of Bcr-Abl tyrosine kinase (Supplementary Figures 1a and b). TKI-resistant K562 cell line was generated by gradual increase in the dose of TKI. The first treatment dose was 0.05 *μ*M TKI, and gradually TKI dose was increased every week. The treatment was continued for 3 months till achievement of 5 *μ*M and the cells were then characterized as TKI-resistant K562 cells. The TKI-resistant cells were then studied for further analysis.

#### Isolation of mononuclear cells from peripheral blood and bone marrow

Bone marrow from CML patients was isolated and mononuclear cells were separated from the whole blood by Ficoll Histo-paque density gradient method. RBCs were lysed from the mononuclear cells by treatment with RBC lysis buffer containing ammonium chloride. The cells were washed with PBS twice. The cells were counted and their viability was checked by the trypan blue method with every thawing. One million mononuclear cells were cultured overnight in a six-well culture plate at 37 °C and 5% CO_2_. This initial 24 h culture helped the cells to revive and expand before they were being subjected to any treatment or used in further experiments.

#### Primary cell culture of peripheral blood cells and bone marrow

Primary cells were cultured in serum free IMDM media supplemented with 1% glutamine (100 mM) and 1% penicillin–streptomycin (100 mM). A five growth factor cocktail comprising 100 ng/ml Flt3-ligand, 100 ng/ml stem cell factor, 20 ng/ml each of interleukin IL-3, IL-6 and granulocyte–macrophage colony-stimulating factor was added to the media. Growth factors were purchased from Peprotech (NJ, USA).

### Small RNA-sequencing

K562—the cell line from a late-stage CML patient expressed BCR-ABL fusion gene b3-a2.^56^ Cells were treated with 0.2 *μ*M concentration of TKI for 72 h. Total RNA was isolated from inhibitor-treated and -untreated cells, and ligated with 3′ and 5′ adapters for small RNAs. The adapter-conjugated RNA was reverse-transcribed with 3′ adapter-specific primers, and amplified by PCR. The PCR products were run on a 8% polyacrylamide gel, 100 bp band was cut and eluted for purification. The purified cDNA from drug-treated cells was given for sequencing on Illumina hiseq platform (San Diego, USA). The miRNA sequence data were mapped on to human genome (version hg18) and mature miRNAs were identified using RNA fold and miRDeep to confirm their stability. Sequences were read as frequency, that is, how many times the miRNA had been read and normalized as TPM (transcripts per million). MiRNAs from drug-treated K562 cells were compared with miRNA data set from untreated K562 cells (Professor Alok Bhattacharya, JNU).

### Gene expression analysis

In brief, total RNA was isolated from CML cells using Trizol method following the manufacturer's instructions. An amount of 20 ng RNA was used to synthesize a specific cDNA of hsa-miRNA182-5p using stem-loop miRNA specific RT primer. qRT-PCR was performed using Sybr green dye (purchased from Kappa, Boston, MA, USA) on Applied Biosystems 7500 fast detection system (Carlsbad, CA, USA). Expression of miRNA was normalized using the expression of the housekeeping genes 18 s r-RNA. Relative quantification of miRNAs was calculated with the delta delta ΔΔCt method. Data are shown as mean of three independent experiments. Error bars represent s.e. of three independent experiments with *P*-values of the mean. *P<0.05, **P<0.01 and ***P<0.001. The Bcr-Abl/Abl ratio was determined for each patient sample (Supplementary Table 1).

### Methylcellulose colony formation assay

K562 cells were counted for cell viability and an equal number of live cells were seeded in 15% FCS with IMDM culture media mixed with 1% methylcellulose. Colonies were determined for their lineage based on morphological characteristics.^57, 58^ Clonal colonies emerged after 3 weeks were counted and scored for contribution to granulocyte, macrophage, erythrocyte and megakaryocyte lineages. The CFU-Es were progenitors with one or two clusters with 10–100 hemoglobinized erythroblasts in each cluster. The BFU-E colonies were characterized as small (3–8 clusters), intermediate (10–16 clusters) or large (>16 clusters) of primitive erythroid progenitors. The CFU-Gs were progenitors of homogeneous population of granulocytes and exhibit the presence of refractive granules in their cytoplasm. The CFU-Ms were clonogenic progenitors of macrophages that display ground glass appearance. The CFU-GEMM displayed heterogeneous population of granulocytes, erythrocyte, macrophage and megakaryocyte. The CFU-GM displayed heterogeneous population of granulocyte and macrophage. Erythroid colonies displayed haemoglobin production by their red brown colour. The granulocytic colonies displayed large refractive granules. Images were taken under fluorescence microscope (Nikon Eclipse TE2000-S; Meville, NY, USA). Colonies of 5–10 different areas were counted per dish. The images were taken at × 4 and × 10. The myeloid cells were calculated by adding CFU-G and CFU-M colony numbers. The erythroid cells were calculated by adding CFU-E and BFU-E colonies. The ME% was used to show a shift in lineage distribution. Data are shown as mean of three independent experiments. Error bars represent as s.e. of three independent experiments with *P*-values of the mean. *P<0.05, **P<0.01 and ***P<0.001.

% of myeloid–erythroid cells (ME%)=((CFU-G+CFU-M)/(CFU-E+BFU-E)) × 100.

### LNA anti-miR

LNA-based oligonucleotides are designed to block miRNA182-5p expression. In LNA oligos, the furanose ring of selected ribose sugars is chemically locked into an RNA-mimicking conformation by the introduction of an O2′, C4′-methylene bridge. The LNA oligos were purchased from GeneX India Biosciences Pvt Ltd (Eurogentec, Belgium).

Scramble Tm-32 °C

5′**-C**AT**CG**TCGATCGTA**GCGC**A-3′

Anti-miRNA182-5p Tm-42 °C

5′-AGTGT**G**AGTTCT**A**C**C**ATTG**CC**AAA-3′

Anti-hsa-let7c-5p Tm-33 °C

5′**-A**A**CC**ATA**C**AAC**C**TA**C**TA**C**CT**CA**-3′. Bold letters represent LNA modification.

The LNA anti-miRs bind to their complementary miRNA and prevent them from binding to their mRNA sequences. Hence, translational block of target mRNA is released in anti-miRNA-transfected cells. Transfection of K562 cells with anti-miRNA182-5p was performed using Lipofectamine2000 as per manufacturer's protocol. Three different concentrations used were unit 1 for 10 nM, 2 for 20 nM and 5 for 50 nM concentration. Scramble oligos were used along with the treatment. After 24 h, cells were cultured with TKI at 0.75 *μ*M for another 48 h. Absorbance was measured after addition of WST1 cell proliferation reagent (Roche, IN, USA) and normalised to 100 after 72 h post transfection of the scramble control. Data are shown as mean of three independent experiments. Error bars represent as s.e. of three independent experiments with *P*-values of the mean. *P<0.05, **P<0.01 and ***P<0.001.

### MiRNA mimics

MiRNA mimics were purchased from Sigma (St. Louis, USA). The transfection of K562 cells was performed according to the manufacturer's protocol. Three different concentrations were used 1 for 10 nM, 2 for 20 nM and 5 for 50 nM concentration. Scramble oligos were used along with the treatment.

### Hes1 knockdown and overexpression

In brief, K562 cells were nucleofected with Hes1 overexpression and knockdown plasmids on Amaxa 4d nucleofector from Lonza and incubated for 3 days. Hes1 was overexpressed by miGR1-Hes1 plasmid. Hes1 knockdown was performed by shRNA against Hes1 open-reading frame. ShRNA-Hes1 plasmids were a kind gift from Dr Adolo Ferrando, Columbia University. MigR1 control and MigR1-Hes1 plasmids were a kind gift from Avinash Bhandoola lab (NIH, USA).

### Flow cytometry

In brief, K562 cells were washed twice in × 1 PBS, fixed in 2% PFA for 10 min at room temperature. Then, cells were permeabilized by Saponin followed by blocking with 5% blotto at room temperature for 30 min. Rabbit anti-Hes1 antibody (Abcam, Cambridge, USA) was incubated as per manufacturer's protocol. Secondary antibody incubation was done at RT for 30 min and washed with PBS. Cells were assessed for Hes1 expression on FACS Calibur flow cytometer (San Jose, USA). The data were analysed using CellQuestPro software (USA) and shown as histograms and dotplots.

### CRISPR-mediated miRNA182 knockout

Designing of *MIR182* constructs—the CRISPR and CRISPR-associated (CAS) system is RNA-based genome-engineering platform. Recently shown *in vitro* reconstitution of the *Streptococcus pyogenes* type II CRISPR system demonstrated that crRNA fused to a normally trans-encoded tracrRNA was sufficient to direct the Cas9 protein to sequence-specifically cleave target DNA sequences matching the crRNA. Here we engineer a single system of this bacterial type II CRISPR system in human cell lines. For designing the guide RNAs (gRNAs), we identified all the NGG sites (represents protospacer adjacent motif sequence) in *MIR182* cluster region ±1000 bp of the target site. To direct Cas9 to cleave sequences of interest, we designed crRNA-tracrRNA fusion oligonucleotides, referred to as gRNAs under the human U6 polymerase III promoter. To identify CRISPR target sites around *MIR182* cluster that should be cleavable without off-target cuts, we therefore examined all 23 bp sequences of the form 5′-GBBBBBBBBBBBBBBBBBBBNGG-3′, where the B's represented the bases at the genome location, for which no sequence of the form 5′-NNNNNNNBBBBBBBBBBBBBNGG-3′ existed at any other location in the human genome.^59^ Identification of CRISPR target sites across *MIR182* locus was done using the Zhang lab tool, MIT. A single plasmid system expressing Cas9, eGFP and GuideRNA was synthesised to target *MIR182* loci (Supplementary Figure 5b). The CRISPR plasmids induced double-strand breaks in HEK293T cells assessed by surveyor nuclease assay represented as indel percentage (Figure 3b). In HEK293 cells, CRISPR plasmids transfection generated deletion of the *MIR182* locus as shown by agarose gel analysis (Supplementary Figure 6a). Each off-target site was assessed for indels. The top five off-targets were chosen based on their quality score and examined for their potential to generate indels Supplementary Figure 8. The off-targets of Cas9+0482 and Cas9+9678 did not reveal indels, whereas Cas9+0425-05 off-target present in the non-coding genomic region induced indels (Figure 3f). A number of 0.5 million K562 cells were nucleofected with CRISPR *MIR182* plasmids and cultured for 72 h. Further, expression of plasmids was monitored by eGFP expression (Supplementary Figure 5c). FACS sorting of 0.5% high eGFP-positive cells was performed on FACS ARIA with an anticipation of higher editing efficiency (Supplementary Figure 5d). A total of 30–50 cells were cultured in a 48-well plate for 10 days and analysed for deletion clones by PCR method. A 1560 bp long DNA sequence of the *MIR182* locus was amplified using *MIR182* genomic locus-specific primers.

### Surveyor mutation detection assay

In brief, HEK293T cells were transfected with CAS9-GFP-GuideRNAs targeting *MIR182* flanking regions. After 48 h, genomic DNA was extracted from the pool of cells, and analysed for Indels using Surveyor mutation detection kit (Transgenomic, Omaha, USA) as per manufacturer's protocol. In brief, PCR amplicons from mutant (test) and wild-type (reference) DNA were hybridized by heating and cooling the mixture to form hetero- and homoduplexes. The annealed heteroduplex/homoduplex mixture was treated with Surveyor Nuclease. DNA fragments were analysed by agarose gel electrophoresis. The formation of new cleavage products due to the presence of one or more mismatches was indicated by the presence of additional bands. The relative size of these cleavage products indicated the location of the mismatch or mismatches (Figure 3a). Intensity of individual DNA bands was measured by ImageJ software. Indel percentage was calculated as per reference protocol.^38, 59^

### Off-target analysis

Off-targets were identified by the Zhang lab, MIT tool. The top five off-targets for each guide RNAs were analysed by the surveyor nuclease mutation detection system.

### Genotype for *MIR182* deletion

*MIR182* genomic site flanking the target sites (1560 bp) was amplified using PCR and was run on a 2% agarose gel containing EtBr. The product length was matched to the expected product and deletion was confirmed by presence of 756, 819 and 1497 bp DNA fragments. Each PCR product was purified using Qiagen PCR purification kit as per manufacturer's instructions and given for Sanger sequencing analysis. Each chromatogram was read using FinchTV software.

### Statistical analysis

Data points are expressed as mean±standard error. Student *t*-test was performed to find the significance of differences. *P* values of less than.05 were considered to be significant.
