# Supplementary material for: MiRNA182 regulates percentage of myeloid and erythroid cells in chronic myeloid leukemia
Source: Cell Death Dis. 2017 Jan 12;8(1):e2547–. doi: 10.1038/cddis.2016.471 (PMC5386378; doi:10.1038/cddis.2016.471)

**Title**

# MiRNA182-5p regulates myeloid-erythroid ratio of chronic myeloid leukemia cells.

Deepak Arya1, 2, Sasikala P.S.1, Cecil Ross3, Dasaradhi Palakodeti4, Shang Li5, Sudhir Krishna1, #

Supplementary File:  Table- 1, Table- 2, Figure S1-12

# Supplementary table 1- Clinical details of CML patients

| Sample | Disease | Response Status | BCR ABL |
|---|---|---|---|
| K562 | CML | Blast | Positive |
| CC1 | CML | Yes | 48.52 |
| CC2 | CML | Yes | Negative |
| CC3 | CML-fresh | Fresh CML | |
| CC4 | CML fresh | Fresh CML | |
| CC5 | CML-chronic | Yes | 48.52 |
| CN1 | CML | No | 10.88 |
| CN2 | CML | No | |
| CN3 | CML-fresh | No | NEG |
| CB1 | CML-BLAST | No | |
| CB2 | CML | Yes | |
| CB3 | CML | | |
| NR1 | CML | No | 0.605 |
| NR2 | CML-NR | No | 57.43 |
| NR3 | CML | No | 25.62 |
| NR4 | CML | No | 0.00354 |
| NR5 | CML | No | |
| NR6 | CML-NR | Yes | |
| NR7 | CML | No | |
| NR8 | CML-blast | No | |

**Supplementary Table 2- Normalised fold change in the expression of miRNA182-5p.**

| MiRNA | Control (Normalised) | Imatinib (Normalised) |
|---|---|---|
| hsa-miR-941 | 1 | 0.068028171 |
| hsa-miR-30c-5p | 1 | 0.720134867 |
| hsa-miR-19a-3p | 1 | 1.267480223 |
| hsa-miR-27b-3p | 1 | 0.084736281 |
| hsa-miR-1246 | 1 | 0.499664526 |
| hsa-miR-642a-3p | 1 | 0 |
| hsa-miR-374b-5p | 1 | 0.072930064 |
| hsa-miR-130b-5p | 1 | 0.008215668 |
| hsa-let-7i-5p | 1 | 0.00079515 |
| hsa-miR-148b-3p | 1 | 0.55020501 |
| hsa-miR-1278 | 1 | 0 |
| hsa-miR-374a-3p | 1 | 0.072611937 |
| hsa-miR-425-5p | 1 | 0.125941609 |
| hsa-miR-24-3p | 1 | 0.086355795 |
| hsa-miR-33a-5p | 1 | 0.001261692 |
| hsa-miR-1291 | 1 | 0.001186051 |
| hsa-miR-15b-5p | 1 | 0.075365058 |
| hsa-let-7d-5p | 1 | 0.00055199 |
| hsa-miR-151a-5p | 1 | 0.039158973 |
| hsa-miR-22-3p | 1 | 0.030651689 |
| hsa-miR-18a-5p | 1 | 0.313138471 |
| hsa-miR-222-3p | 1 | 0 |
| hsa-miR-144-3p | 1 | 0.008879411 |
| hsa-miR-320b | 1 | 0.003410919 |
| hsa-miR-151a-3p | 1 | 0.103428327 |
| hsa-miR-330-3p | 1 | 0.008772168 |
| hsa-miR-125a-5p | 1 | 0.049376705 |
| hsa-miR-182-5p | 1 | 2.998313088 |
| hsa-let-7b-5p | 1 | 0.000369092 |
| hsa-miR-23a-3p | 1 | 0.103891736 |
| hsa-miR-148a-3p | 1 | 0.630124942 |
| hsa-miR-7-5p | 1 | 0.093070343 |
| hsa-miR-744-5p | 1 | 0.000901427 |
| hsa-miR-99b-5p | 1 | 0.101314617 |
| hsa-miR-92b-3p | 1 | 0.002587935 |
| hsa-miR-486-3p | 1 | 0.004077553 |
| hsa-miR-106b-5p | 1 | 0.053452099 |
| hsa-miR-423-3p | 1 | 0.014604611 |
| hsa-miR-221-3p | 1 | 0 |
| hsa-miR-16-5p | 1 | 0.180895997 |
| hsa-miR-186-5p | 1 | 0.089614021 |
| hsa-let-7c | 1 | 0.003299537 |
| hsa-miR-223-3p | 1 | 0.005871449 |

| | | |
|---|---|---|
| hsa-miR-93-5p | 1 | 0.449292322 |
| hsa-miR-340-5p | 1 | 0.005942312 |
| hsa-miR-144-5p | 1 | 0.478218307 |
| hsa-miR-26a-5p | 1 | 0.032706767 |
| hsa-miR-29a-3p | 1 | 0.01236458 |
| hsa-miR-30e-5p | 1 | 0.712356005 |
| hsa-miR-26b-5p | 1 | 0.008254059 |
| hsa-miR-128 | 1 | 0.002599649 |
| hsa-miR-224-5p | 1 | 0.002482631 |
| hsa-miR-142-5p | 1 | 0.006297749 |
| hsa-miR-486-5p | 1 | 0.063843255 |
| hsa-miR-130b-3p | 1 | 0.023893347 |
| hsa-miR-20a-5p | 1 | 0.664997979 |
| hsa-miR-10a-5p | 1 | 0.120275996 |
| hsa-miR-451a | 1 | 0.493771868 |
| hsa-miR-17-5p | 1 | 0.226033823 |
| hsa-miR-107 | 1 | 0.000908133 |
| hsa-let-7g-5p | 1 | 0 |
| hsa-miR-17-3p | 1 | 0.004599515 |
| hsa-miR-192-5p | 1 | 0.020082504 |
| hsa-miR-452-5p | 1 | 0.010758428 |
| hsa-miR-30d-5p | 1 | 0.086356953 |
| hsa-miR-19b-3p | 1 | 0.132467871 |
| hsa-miR-25-5p | 1 | 0.000126266 |
| hsa-miR-146b-5p | 1 | 0.091046425 |
| hsa-miR-101-3p | 1 | 0.066583124 |
| hsa-miR-191-5p | 1 | 0.018408132 |
| hsa-miR-378a-3p | 1 | 0.450659093 |
| hsa-miR-92a-1-5p | 1 | 0.00012145 |
| hsa-let-7e-5p | 1 | 0.00075418 |
| hsa-miR-25-3p | 1 | 0.004684743 |
| hsa-miR-140-3p | 1 | 0.0102982 |
| hsa-miR-21-5p | 1 | 0.023982188 |
| hsa-miR-423-5p | 1 | 0.00626765 |
| hsa-miR-92a-3p | 1 | 0.015070228 |
| hsa-miR-103a-3p | 1 | 0.016940454 |
| hsa-let-7f-5p | 1 | 0.001901265 |
| hsa-miR-185-5p | 1 | 0.001187326 |
| hsa-let-7a-5p | 1 | 0.000560282 |
| hsa-miR-320a | 1 | 0.000452567 |

Fig.S1 Effect of Imatinib on Bcr-Abl tyrosine kinase.

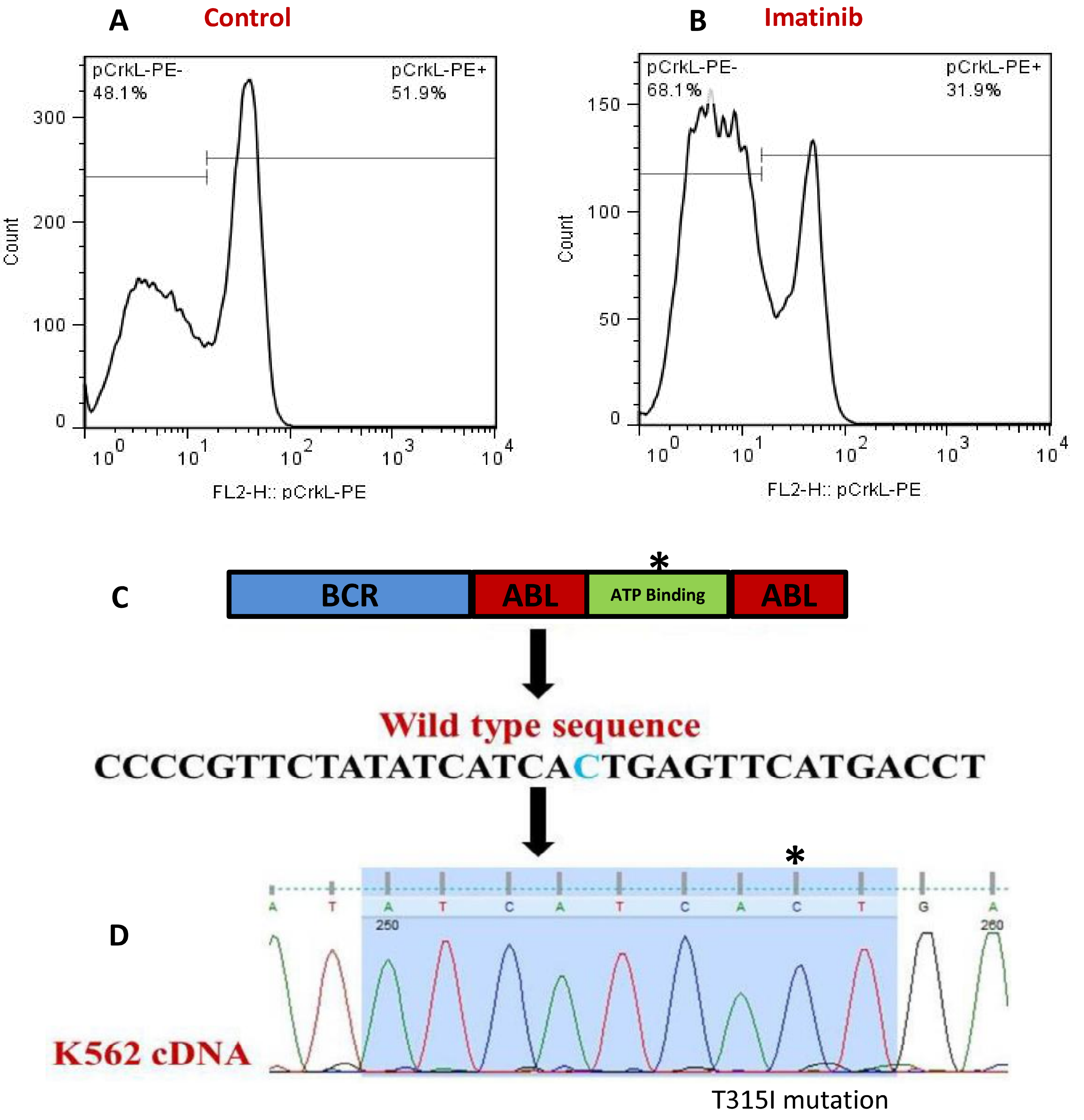

# Fig.S2 MiRNA182-5p negatively correlates with Notch signalling target genes in the context of TKI resistance.

A

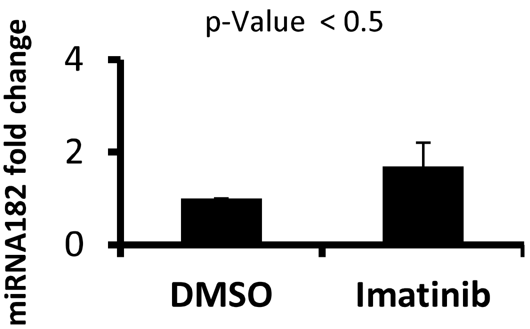


B

| 551 | hsa-miR-182-5p | HES1 (ENSG00000114315) | 0.867198 |
|---|---|---|---|

**Gene Description:** TRANSCRIPTION FACTOR HES-1 (HAIRY AND ENHANCER OF SPLIT 1)(HAIRY-LIKE)(HHL)(HAIRY HOMOLOG) [SOURCE:UNIPROTKB/SWISS-PROT;ACC:Q14469]
**PubMed Links:** miRNA | gene | both
**KEGG Pathways:** Maturity onset diabetes of the young
Notch signaling pathway
**Gene Links:** UniProt

ENST00000232424

| Region | Binding Type | Position | Score | Conservation |
|---|---|---|---|---|
| UTR3 | 7mer | 285 - 293 | 0.0413736 | 13 |

**Position on chromosome:** 3 : 195338982 - 195339010
**Conserved species:** panTro2,rheMac2,rn4,mm8,oryCun1,bosTau3,loxAfr1,echTel1,monDom4,xenTro2,tetNig1,fr2,danRer4
**RNA hybrid:**

```
(3' UTR) 5'AC    AU     UUU     GUGA        3'
           GAG  UUCU   UUAU    UGCCAAA
           |||  ||||   ..||    |||||||
           CUC  AAGA   GGUA    ACGGUUU
(miRNA)  3' A          U                  5'
```

C

**Conserved**

| | predicted consequential pairing of target region (top) and miRNA (bottom) | seed match | site-type contri-bution | 3' pairing contri-bution | local AU contri-bution | position contri-bution | TA contribution | SPS contribution | context+ score | context+ score percentile | conserved branch length | $P_{CT}$ |
|---|---|---|---|---|---|---|---|---|---|---|---|---|
| Position 288-294 of HES1 3' UTR<br>hsa-miR-182 | 5' ...UUCUUUUUUAUGUGAUGCCAAAG...<br>\|\|\|\|\|\|<br>3' UCACACUCAAGAUGGUAACGGUUU | 7mer-1A | -0.074 | 0.001 | -0.011 | -0.041 | 0.010 | -0.027 | -0.14 | 70 | 2.419 | 0.74 |

D

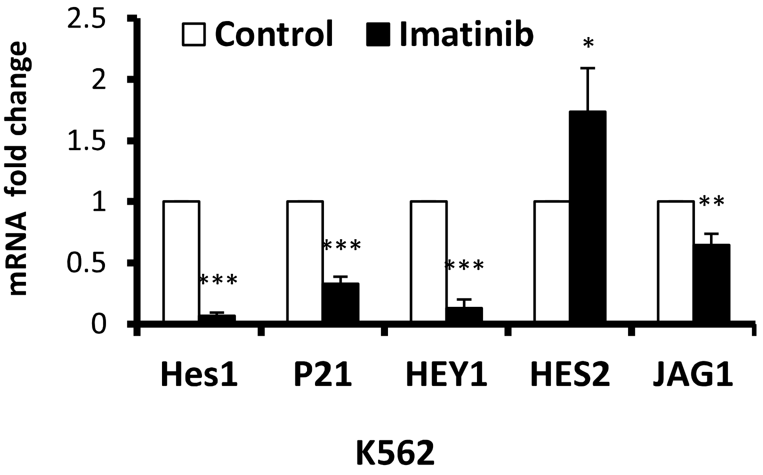


E

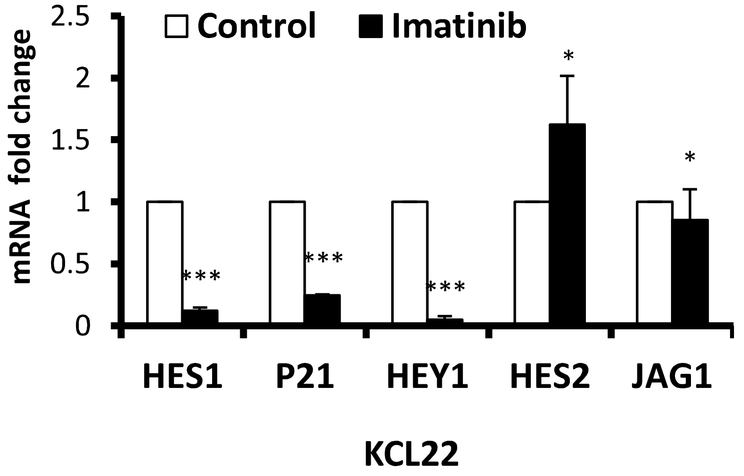

# Fig.S3 Analysis of miRNA182 signalling components.

**A**

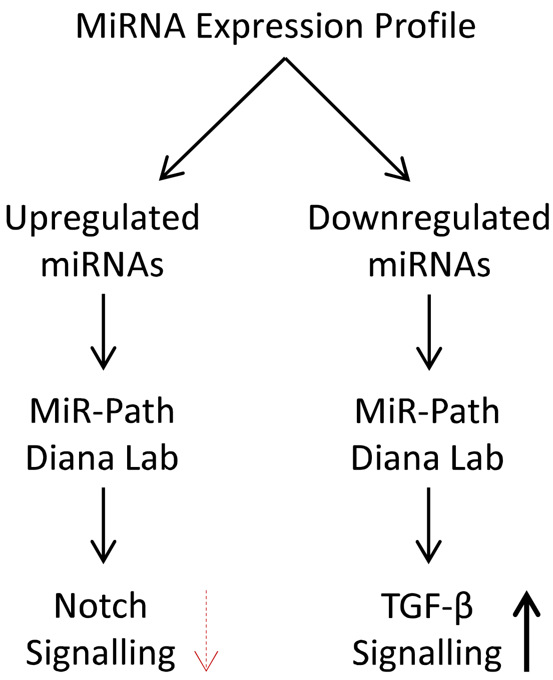


**B**

| # | KEGG pathway | p-value | #genes | | #miRNAs |
|---|---|---|---|---|---|
| 1. | Fatty acid biosynthesis (hsa00061) | <1e-16 | 1 | see genes | 2 |
| 2. | ECM-receptor interaction (hsa04512) | <1e-16 | 12 | see genes | 6 |
| 3. | Glycosaminoglycan biosynthesis - heparan sulfate (hsa00534) | <1e-16 | 7 | see genes | 7 |
| 4. | Mucin type O-Glycan biosynthesis (hsa00512) | 1.776357e-15 | 7 | see genes | 5 |
| 5. | TGF-beta signaling pathway (hsa04350) | 1.133326e-09 | 35 | see genes | 2 |
| 6. | Prion diseases (hsa05020) | 4.753938e-08 | 2 | see genes | 1 |
| 7. | Lysine degradation (hsa00310) | 3.177965e-05 | 3 | see genes | 1 |
| 8. | Axon guidance (hsa04360) | 7.888913e-05 | 27 | see genes | 4 |
| 9. | Endocytosis (hsa04144) | 0.0003580835 | 57 | see genes | 4 |

**C** **D** **E**

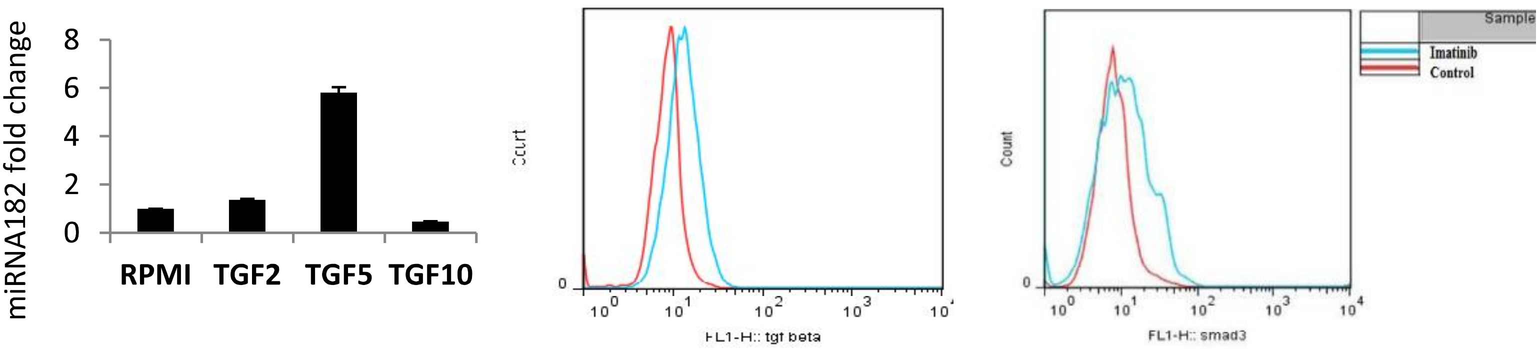

**Fig.S4 Effect of anti-miRNA182-5p on Hes1 expression in primary CML cells.**

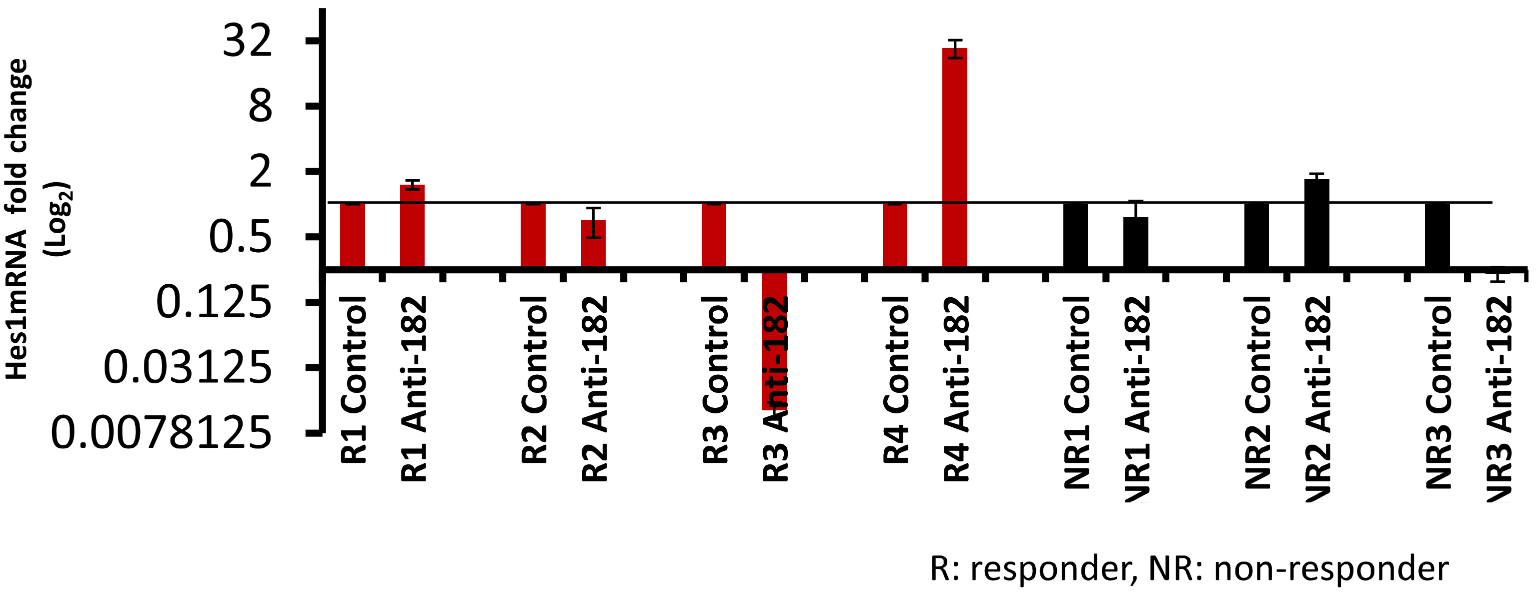

# Fig.S5 Experimental layout of *MIR182* knockout in K562 cells.

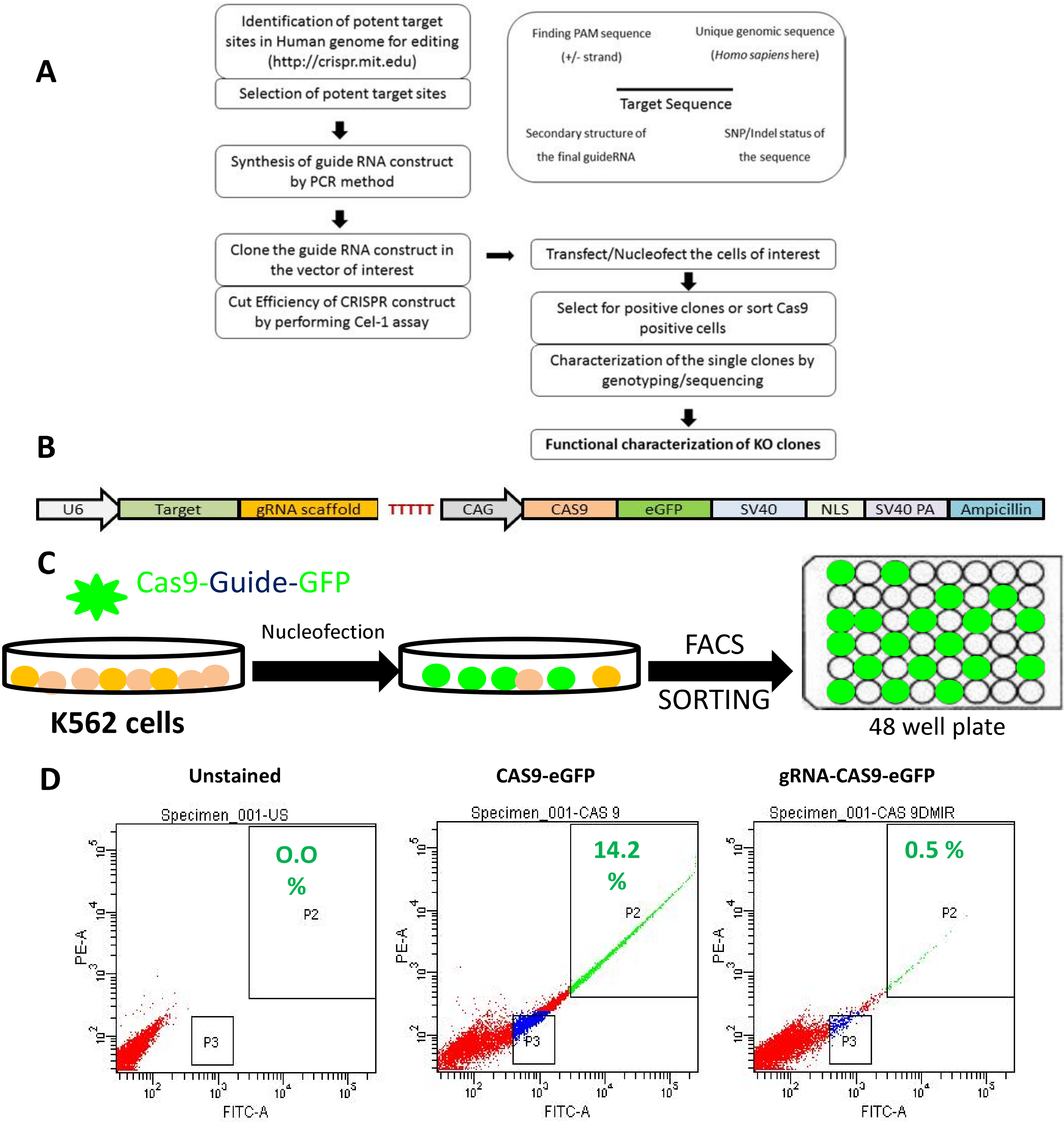

**Fig.S6 CRISPR mediated *MIR182* knockout in HEK293 cells.**

**A**

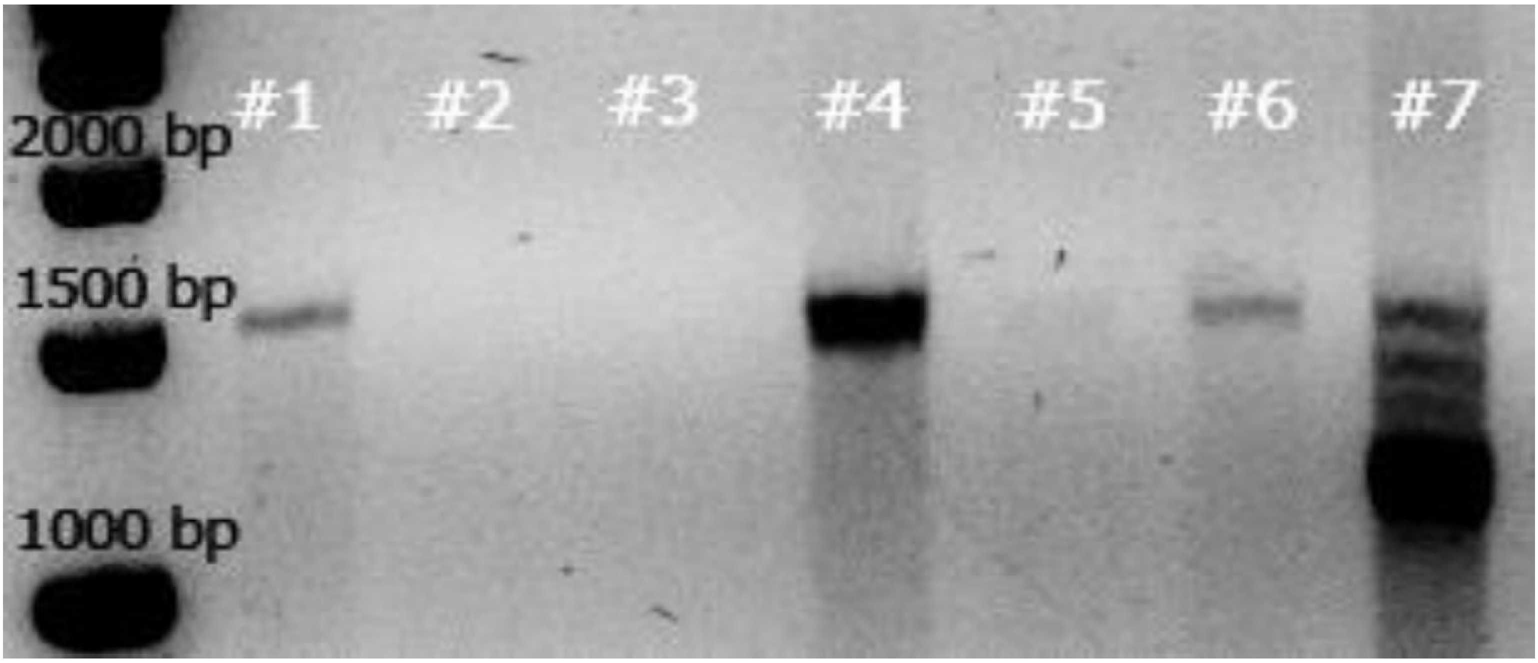


**B**

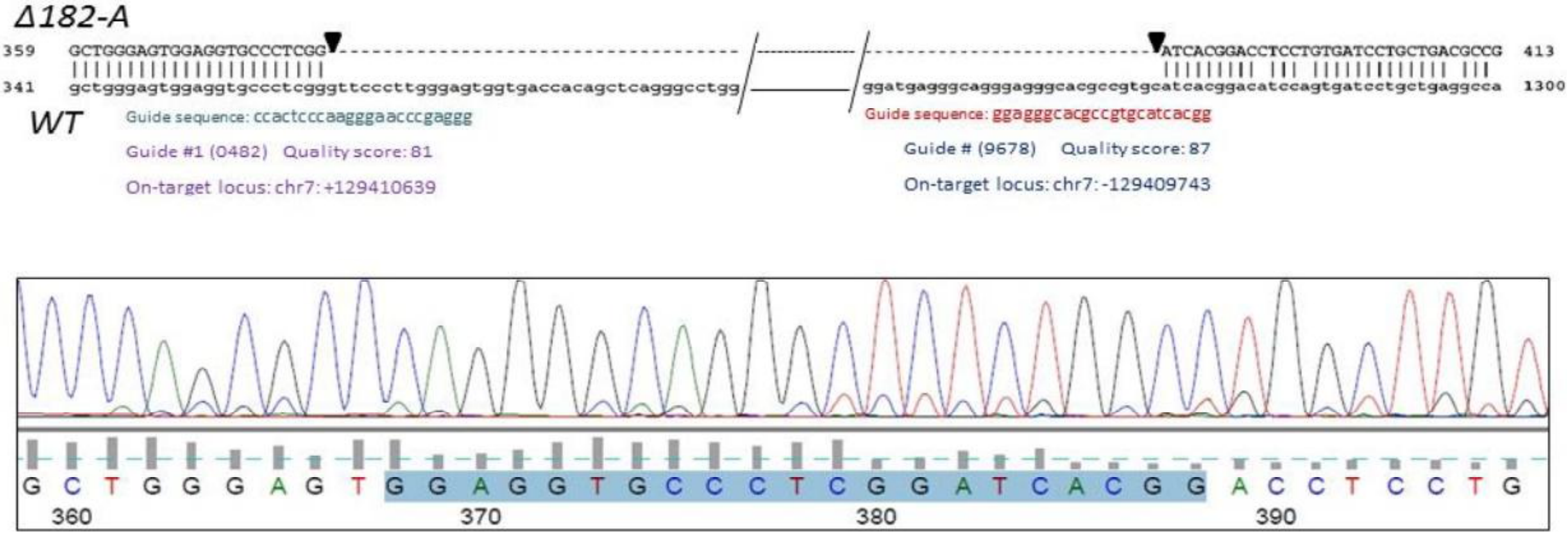

**Fig.S7 Effect of modulation in expression of miRNA182-5p on differentiation potential of K562 cells.**

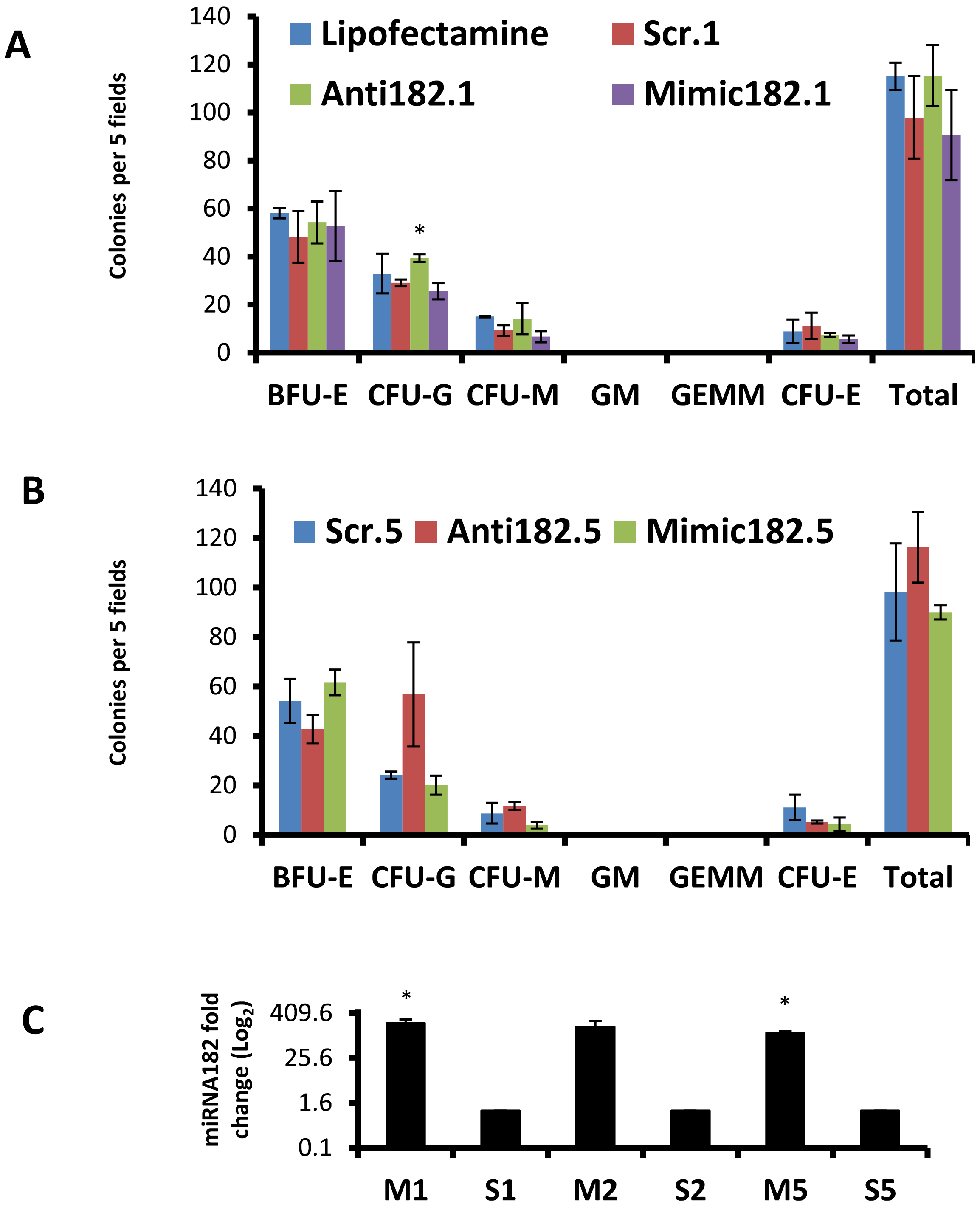

# Fig.S8 Off-target analysis of CRISPR guideRNAs targeting *MIR182* locus.

## Guide #4 (0425)

**Quality score:** 88

**Guide sequence:** GAAGGACCTTGTCGCAGTTG**CGG**

**On-target locus:** chr7:-129410425

**Number of off-target sites:** 90 (25 are in genes)

| Sequence | score | mismatches | UCSC gene | locus |
|---|---|---|---|---|
| TGAAGGCCTTGTCGCAGTTGGGG | 0.8 | 4MMs [1:2:4:6] | NM_020807 | chr16:+58030598 |
| TAAAAACCTTGTGGCAGTTGCAG | 0.6 | 4MMs [1:4:5:13] | | chr3:-106872028 |
| GTAGAACGTTGTTGCAGTTGTAG | 0.6 | 4MMs [2:5:8:13] | | chr8:+83102115 |
| GTAGGCTCTCGTCGCAGTTGAAG | 0.5 | 4MMs [2:6:7:10] | NR_029427 | chrX:-48463337 |
| GCAGGACCTTGTTGCAATTGAAG | 0.5 | 3MMs [2:13:17] | | chr5:-27227006 |

## Guide #1 (0482)

**Quality score:** 81

**Guide sequence:** CCACTCCCAAGGGAACCCGA**GGG**

**On-target locus:** chr7:+129410639

**Number of off-target sites:** 145 (15 are in genes)

| sequence | score | mismatches | UCSC gene | locus |
|---|---|---|---|---|
| TGACACCCAGGGGAACCCGATGG | 1.3 | 4MMs [1:2:5:10] | | chr10:+72837071 |
| CTGCTCTGAAGGGAACCCGAGGG | 0.9 | 4MMs [2:3:7:8] | | chr7:-47843799 |
| TGACTCCAGAGGGAACCCGAAAG | 0.9 | 4MMs [1:2:8:9] | | chr17:+3868097 |
| CCAAACCTCAGGGAACCCGAGGG | 0.8 | 4MMs [4:5:8:9] | | chr10:+98478197 |
| CCAATCCATCGGGAACCCGAGGG | 0.8 | 4MMs [4:8:9:10] | | chr6:-30449530 |

## Guide #2 (9678)

**Quality score:** 87

**Guide sequence:** GGAGGGCACGCCGTGCATCA**CGG**

**On-target locus:** chr7:-129409743

**Number of off-target sites:** 83 (22 are in genes)

| sequence | score | mismatches | UCSC gene | locus |
|---|---|---|---|---|
| GGAGGGCAGGCCGTGCATCCAGG | 2.4 | 2MMs [9:20] | | chr20:+43221228 |
| CCAGGGCAGTCCGTGCATCAGAG | 0.8 | 4MMs [1:2:9:10] | | chr17:+48274952 |
| GGAGGCCTCGCCGTGCGTCACAG | 0.7 | 3MMs [6:8:17] | NM_001040285 | chr16:-50187013 |
| GGCTTGCACGCCCTGCATCAAAG | 0.6 | 4MMs [3:4:5:13] | | chr17:+6113729 |
| GGAGGGCAGTCCCTGCATCAGGG | 0.5 | 3MMs [9:10:13] | | chr11:-134043507 |

**Fig.S9 TKI resistance is associated with a shift in ME% of K562 cells.**

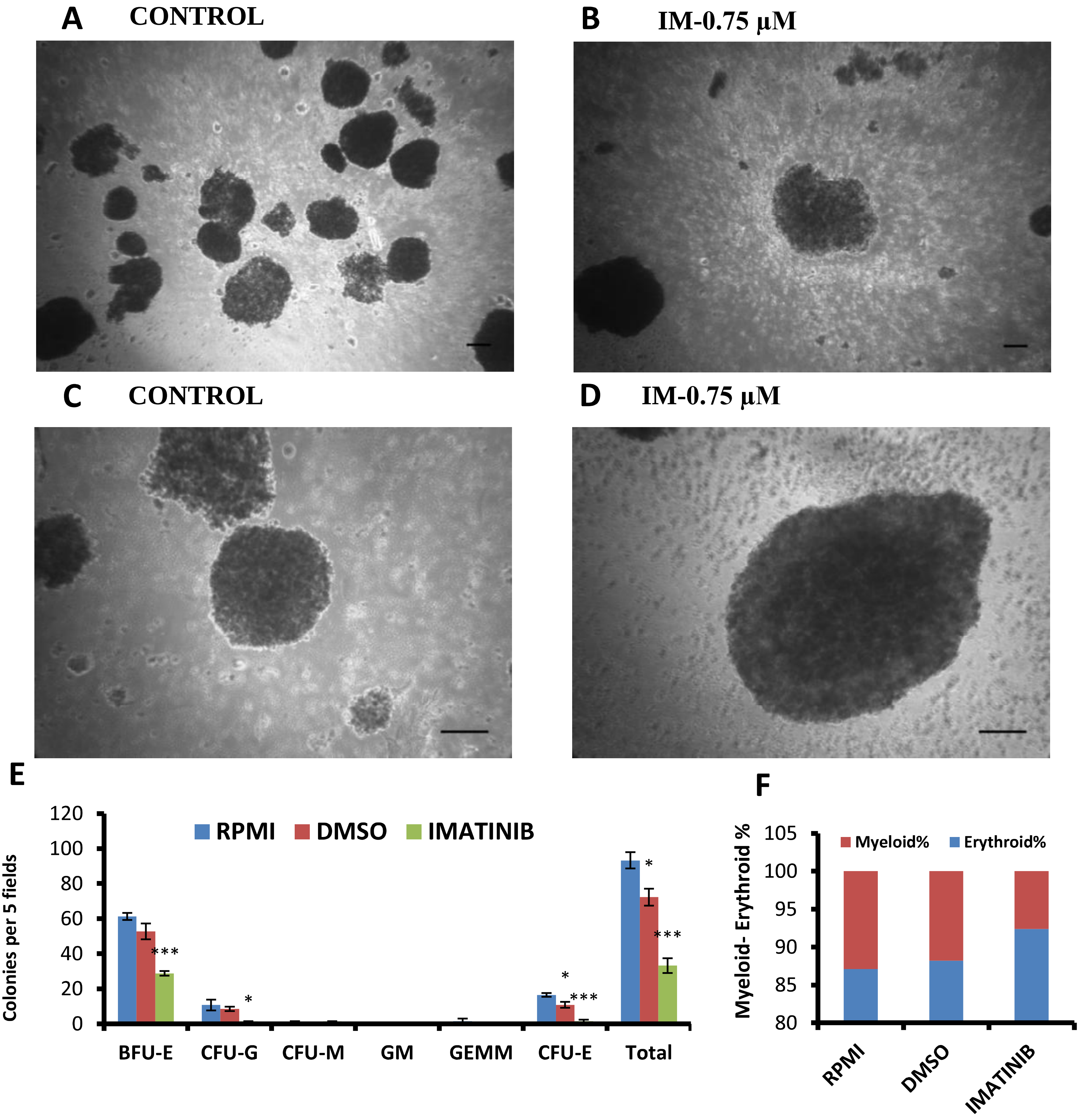

**Fig. S10 Proliferation potential of *Δ182* cells.**

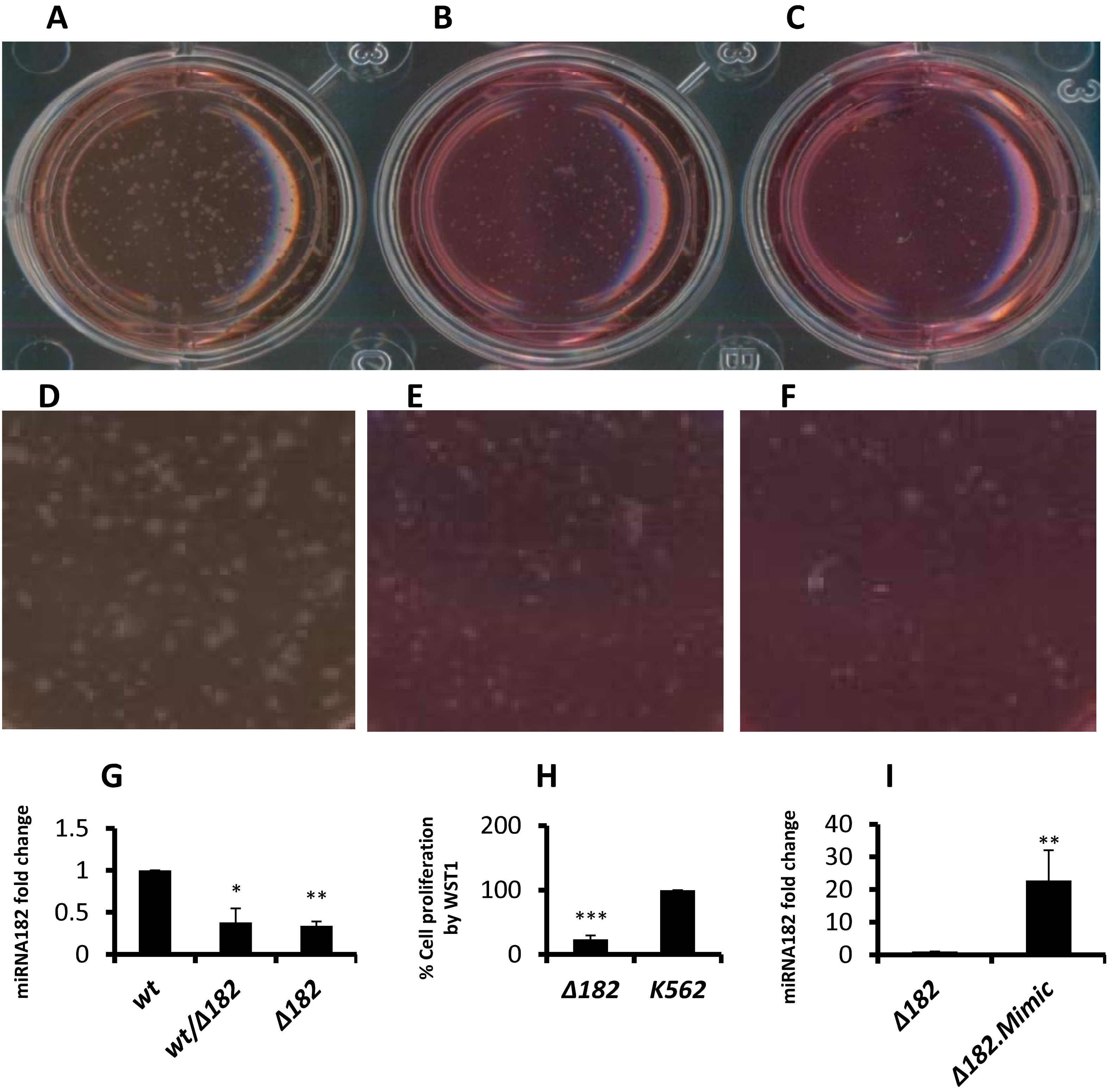

Supplement: Supplementary Material [file cddis2016471x1.pdf]
